# Tunable Neural Encoding of a Symbolic Robotic Manipulation Algorithm

**DOI:** 10.3389/fnbot.2021.744031

**Published:** 2021-12-14

**Authors:** Garrett E. Katz, Akshay, Gregory P. Davis, Rodolphe J. Gentili, James A. Reggia

**Affiliations:** ^1^Department of Electrical Engineering and Computer Science, Syracuse University, Syracuse, NY, United States; ^2^Department of Computer Science, University of Maryland, College Park, MD, United States; ^3^Department of Kinesiology, University of Maryland, College Park, MD, United States

**Keywords:** neurosymbolic architectures, robotic manipulation, reinforcement learning, policy optimization, explainable AI

## Abstract

We present a neurocomputational controller for robotic manipulation based on the recently developed “neural virtual machine” (NVM). The NVM is a purely neural recurrent architecture that emulates a Turing-complete, purely symbolic virtual machine. We program the NVM with a symbolic algorithm that solves blocks-world restacking problems, and execute it in a robotic simulation environment. Our results show that the NVM-based controller can faithfully replicate the execution traces and performance levels of a traditional non-neural program executing the same restacking procedure. Moreover, after programming the NVM, the neurocomputational encodings of symbolic block stacking knowledge can be fine-tuned to further improve performance, by applying reinforcement learning to the underlying neural architecture.

## 1. Introduction

Effective manipulation requires tight integration of low-level motor control and high-level reasoning. In robotics and AI, high-level reasoning is usually implemented using symbolic methods, such as automated first-order logic and back-tracking search (Russell and Norvig, 2002; Ghallab et al., 2004), whereas low-level motor control uses sub-symbolic methods such as neural networks (Gentili et al., 2015; Levine et al., 2016).

Integrating symbolic reasoning with sub-symbolic robotic control, also known as “Cognitive Robotics” (Levesque and Lakemeyer, 2008), is a long-standing challenge and active research area. Symbolic methods make it straightforward for human engineers to specify declarative and procedural knowledge and goals, but do not readily handle raw sensorimotor data, adapt to changing environments, or learn from experience to improve performance. The situation with neural networks and other sub-symbolic control methods is reversed. Hence, there is a need for robotic systems that tightly integrate both methodologies and leverage the best of both worlds.

Programmable neural networks (Verona et al., 1991; Neto et al., 2003; Eliasmith and Stewart, 2011; Bošnjak et al., 2017; Katz et al., 2019; Davis et al., 2021) comprise one potential approach to building such systems. These are neural networks whose dynamics can emulate execution of human-authored source code. Typically, each symbol in the source code is represented by a dedicated pattern vector of neural activity, and different layers of neurons represent different registers and memory slots in the symbolic machine being emulated. A mathematical construction then converts the source code to an equivalent set of synaptic weight values that are assigned to the neural network, analogously to a “compilation” process. Finally, one runs the resulting network activation dynamics, and changing pattern vectors in the various neural layers correspond one-to-one with changes in the emulated symbolic machine state during program execution.

In a robotics context, a human operator can write a symbolic program for a manipulation task, and then “compile” this program into an equivalent programmable neural network instance. This can be viewed as a sophisticated form of weight initialization that encodes prior declarative and procedural knowledge. The benefit is that this prior knowledge could be used as inductive bias and fine-tuned over time to improve performance, by applying sub-symbolic learning techniques to the underlying neural network as it interacts with the environment. As opposed to training black-box neural networks from scratch, this may produce more explainable AI systems, due to the encoded prior knowledge used as a starting point.

In this paper, we test this approach using a programmable neural network model called the “Neural Virtual Machine” (NVM), which is asymptotically Turing-complete as layer size grows (Katz et al., 2019). We program the NVM to control the Poppy™ Ergo Jr robotic manipulator (Lapeyre et al., 2014) in a PyBullet (Coumans and Bai, 2021) simulation environment. The code for all experiments is open-source and available online.^1^ Our focus in this paper is not state-of-the-art low-level motor control, nor is it state-of-the-art high-level planning. Rather, it is state-of-the-art methodology for integration of symbolic and sub-symbolic aspects of robotic manipulation using programmable neural networks. To that end, we employ a simple block stacking task and planning algorithm. This avoids the finer points of motor control and automated planning research, when considered separately, that are not germane to our study.

## 2. Background

### 2.1. Block Stacking

Block stacking is a classic problem domain in AI and robotics (Nilsson, 1980; Russell and Norvig, 2002; Ghallab et al., 2004), where the goal is to rearrange stacks of blocks into a target configuration by picking up or putting down blocks, one at a time. Despite the simplicity of the problem statement, computing an optimal sequence of pick-up and put-down actions is computationally complex. Gupta and Nau (1991) show that a common formalization of optimal blocks-world planning is NP-hard, due to a situation they call “deadlock” in which two (or more) blocks cover each other's goal positions. Another well-known issue in block stacking and other planning domains is “Sussman's anomaly,” in which premature resolution of one sub-goal may prevent another sub-goal from being achieved without undoing the first (Sussman, 1973). Many sophisticated planners avoid these issues and perform well on block stacking in the average case.

If one is not concerned with *optimal* block stacking, one can achieve the goal in a slower but straightforward way: first unstack every block until all blocks are flat on the tabletop, and then stack blocks back up into the desired arrangement. This procedure avoids Sussman's anomaly because it does not prematurely stack one block on another until after all blocks are laid out on the table. Since our focus here is integrating symbolic and neurocomputational robotic control, rather than optimal planning at the purely symbolic level, we adopt this simpler but sub-optimal block stacking procedure.

### 2.2. Programmable Neural Networks

The question of how neural networks (including the human brain) can support cognitive-level symbolic processing is a long-standing research problem. One approach to this problem aims to “compile” symbolic source code into a set of equivalent neural network weights, such that running the resulting network dynamics effectively emulates execution of the source code. We refer to such models as “programmable neural networks.” Examples from the past several decades include (Verona et al., 1991; Gruau et al., 1995; Neto et al., 2003; Eliasmith and Stewart, 2011), which often use local representation (i.e., one neuron represents one program variable) and/or static weights that do not change after “compilation” time. More recent approaches often use modern deep learning tools and gradient-based optimization to obtain the weights from training examples (Graves et al., 2016; Reed and De Freitas, 2016; Bošnjak et al., 2017), and employ model architectures that are “hybrid” (not purely neural) or otherwise biologically implausible.

Programmable neural networks are one potential route to cognitive robotics, because they can represent symbolic knowledge but are also amenable to sub-symbolic processing. An early example of neural network control in manipulation tasks comes from Dehaene and Changeux (1997), who used local representation and hand-crafted weights to perform the Towers of London stacking task. More recently, Aleksander (2004) used a neural network to generate mental imagery of a plan to restack blocks, although no robotic manipulation was included. A recent deep learning approach by Xu et al. (2018) is capable of robotic imitation learning, although it wraps neural components in a symbolic top-level control algorithm.

### 2.3. Contributions

In this paper, we present a programmable neural network approach capable of encoding symbolic procedural knowledge for block stacking, and executing the encoded block stacking algorithm on a simulated robotic manipulator. Unlike past programmable neural networks, ours uses a purely neural architecture, distributed representations, and dynamic weights that change during both program “compilation” and program execution. Whereas past work also focuses only on compilation or only on learning, we demonstrate that our model supports both: it can compile human-authored procedural knowledge into initial network weights, but also refine that knowledge by fine-tuning the weights on the basis of experience and reinforcement signals.

Our programmable neural network used here is an updated version of our recently proposed “Neural Virtual Machine” (NVM) architecture (Katz et al., 2019). The following section provides the requisite background information needed to understand the NVM and how we use it here.

### 2.4. The Neural Virtual Machine

The NVM is a programmable neural network that emulates a virtual, symbolic machine. The virtual machine (VM) can execute programs written in a minimalistic, assembly-like language. There is also a non-neural, reference implementation of this virtual machine, which we will call the RVM. The RVM is a purely symbolic implementation of the VM, so it does not suffer from numerical issues faced by neurocomputational systems. Therefore, RVM execution traces serve as a “gold standard,” against which the NVM can be compared for testing and validation purposes.

**Figure 1:**
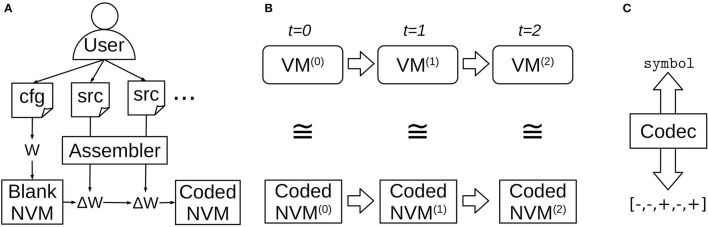
Top-level NVM workflow. **(A)** The user configures a blank NVM instance and then compiles one or more assembly source programs into weight updates. **(B)** Running the recurrent NVM dynamics produces network states in one-to-one correspondence with the emulated virtual machine states. **(C)** At any point the user may employ a lookup table called a “codec” to convert between layer activity patterns and the symbols they represent, in order to inspect the emulated machine state or supply input.

The top-level NVM workflow is shown in Figure 1. First, a user provides a desired NVM configuration, including layer sizes and any application-specific layers and connections (Figure 1A). A “blank” (not yet programmed) NVM instance is automatically constructed with the desired configuration and appropriate initial weights *W*. Then, the user can supply source code for one or more programs in the VM assembly language. Distinct, random patterns of neural activity are assigned to represent different symbols appearing in the source code. Each program is then “compiled” by the NVM assembler into a weight update Δ*W* that encodes the program and is applied to the NVM instance.

A compiled program can be executed by running the recurrent NVM dynamics (Figure 1B). The NVM neural network state at each time-step is in one-to-one correspondence with the VM machine state that it represents. Each virtual machine register has a corresponding neural layer, and the layer's activity pattern represents the symbol currently stored in the register.

The assignments of activity patterns to symbols are stored in a bidirectional lookup table called a “codec,” because it can “encode” a symbol by looking up its assigned pattern, or “decode” a pattern by looking up the symbol it represents (Figure 1C). The codec can be used at any point to encode and inject symbolic input, or extract and decode symbolic output.

We provide more detail on the symbolic VM, and then the neural network that emulates it, in the following two sections. Later, section 3.3 provides a concrete example of an NVM execution trace to illustrate how the various layers and weights work together to emulate a full program relevant to block stacking.

#### 2.4.1. The Reference VM

The RVM contains a set of registers and a set of directed connections between those registers. Each register can hold one symbol at a time. A symbol currently held in a register r is denoted *v*_r_. There can be multiple connections between the same pair of registers, and self-connections from a register to itself. Each connection operates like a rewritable, key-value lookup table, where symbols can be used as keys or values. Given a connection C from a source register q to a destination register r, two fundamental operations can be performed:

- Storage of a key-value pair: C[*v*_q_] ← *v*_r_
- Recalling a value from its key: *v*_r_ ← C[*v*_q_]

Storing a new key-value pair in a connection will overwrite any previously stored pair with the same key. Connections have similar semantics to mapping types in high-level programming languages, such as dictionaries in Python, except that they are bound to specific pairs of registers. The connection C above can only store a new key-value pair if the key and value are currently in q and r, respectively. Likewise, it can only recall a value for the key currently in q, and the value it recalls will overwrite the content of r.

VM connections can be used to store domain-specific mapping data (e.g., mapping blocks to their locations), but they can also be used in more versatile ways to implement generic assembly language constructs. The simplest example is copying data between registers. If a connection between registers maps each possible symbol *v* to itself, i.e., C[*v*] = *v*, then recall in the connection will copy the current symbol in the source register to the destination register.

**Figure 2:**
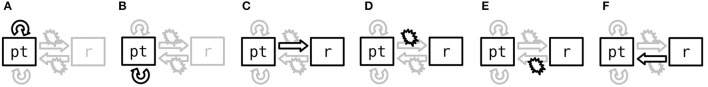
Representing random-access memory operations with storage and recall gating patterns in NVM connections. Boxes denote register layers. Block arrows and explosion symbols indicate recall and storage, respectively, in connections between layers. The performed (ungated) operation and the layers involved are shown in black. Adapted from Katz et al. (2019). **(A)** Increment. **(B)** Decrement. **(C)** Read. **(D)** Write. **(E)** Reference. **(F)** Dereference.

A more complex but powerful example is representing random-access memory, with reading, writing, pointer arithmetic, and pointer reference/dereferencing. This can be achieved with two registers and four connections, as shown in Figure 2. Symbols in one register, called pt in the figure, represent memory address pointers. The pt register functions like a read-write head. Symbols in the other register, called r in the figure, represent generic symbols that can be written to/read from memory, or to/from which pointers can be referenced/dereferenced. As shown in the figure, each memory operation can be implemented by specific storage or recall operations in the appropriate connections. In more detail:

- Pointer increments use a connection C_inc_ from pt to itself. If the *i*^*th*^ memory address is represented by a symbol m_*i*_, and C_inc_[m_*i*_] = m_*i*+1_ for each *i*, then recall in C_inc_ increments the current pointer in pt. Similarly, decrements use another self-connection C_dec_ in which C_dec_[m_*i*_] = m_*i*−1_.
- Memory reading and writing uses the connection C_pt, r_ from pt to r. The storage operation C_pt, r_[*v*_pt_] ← *v*_r_ writes the symbol in r to the memory address in pt, and the recall operation *v*_r_ ← C_pt, r_[*v*_pt_] reads a symbol from the memory address in pt into register r.
- Pointer de/reference uses the reverse connection C_r, pt_, from r to pt. Storage will associate the current symbol in r with the current address in pt (pointer reference), and recall will dereference the current symbol in r, restoring its associated address in pt.

The foregoing mechanisms are not limited to programmer-facing heap memory. They can also be used for program memory with instruction pointers, and stack frame memory for sub-routine calls with stack pointers. Therefore, VM connections are a quite general abstraction that supports random-access memory and non-sequential program execution.

#### 2.4.2. The Neural VM

The NVM is a purely neurocomputation implementation of the reference VM described above. Each register is represented by a layer of neurons, each possible symbol is represented by a distinct, dedicated pattern vector, and each connection is represented by an associative weight matrix. The overall network is recurrent, so neural activities in each layer and synaptic weights in each connection change over time. The current activity pattern in a layer represents the current symbol in the corresponding register.

The connection recall operation, *v*_r_ ← C[*v*_q_], is implemented by one time-step of associative recall with the corresponding weight matrix:


(1) $$\begin{array}{l} {\text{v}}_{\text{r}}^{\left(t+1\right)}=\sigma \left(W_{\text{C}}^{\left(t\right)}{\text{v}}_{\text{q}}^{\left(t\right)}\right), \end{array}$$

where **v**^(*t*)^ and *W*^(*t*)^ denote neural activity vectors and weight matrices at time-step *t*, and σ is a suitable element-wise activation function (see Equation 5 below). The connection storage operation, C[*v*_q_] ← *v*_r_, is implemented by one time-step of associative learning in the corresponding weight matrix:


(2) $$\begin{array}{l} W_{\text{C}}^{\left(t+1\right)}=W_{\text{C}}^{\left(t\right)}+\Delta W_{\text{C}}^{\left(t\right)} \end{array}$$

(3) $$\begin{array}{l} \Delta W_{\text{C}}^{\left(t\right)}=H\left(W_{\text{C}}^{\left(t\right)},{\text{v}}_{\text{q}}^{\left(t\right)},{\text{v}}_{\text{r}}^{\left(t\right)}\right) \end{array}$$

where $\Delta W_{\text{C}}^{\left(t\right)}$ is a one-shot, fast weight update to $W_{\text{C}}^{\left(t\right)}$. This weight update $\Delta W_{\text{C}}^{\left(t\right)}$ is a function *H* of the current connection weights and activities in source and destination layers. The NVM uses the following “fast store-erase learning rule” for the function *H*:


(4) $$\begin{array}{l} H\left(W,\text{x},\text{y}\right)=\left(\text{y}-W\text{x}\right){\text{x}}^{⊤}/N, \end{array}$$

where **x** represents the key, **y** represents the value, **x**^⊤^ denotes transpose, and *N* is the number of neurons in pattern **x**. This learning rule combines Hebbian and anti-Hebbian terms (Hebb, 1949) to simultaneously add new key-value associations and overwrite old ones when appropriate.

It has been shown that these recall and storage rules perfectly replicate the semantics of a symbolic key-value lookup table, as long as certain conditions are met (Katz et al., 2019). Specifically, distinct key symbols must be represented by orthogonal pattern vectors with ±1 elements. Such sets of patterns vectors can be computed via Sylvester's Hadamard matrix construction (Sylvester, 1867). In the presence of small noise or accumulating round-off error over time, we can ensure that ±1 patterns are preserved with the element-wise activation function


(5) $$\begin{array}{l} \sigma \left(a\right)=\tanh\left(a\right)/\tanh\left(1\right), \end{array}$$

which has stable fixed points at *a* = ±1.

When the VM (and NVM) execute a program, not all connections should be storing and recalling simultaneously. Only a small subset of connections should be storing or recalling at a given time, as illustrated in Figure 2. Moreover, when a register r has multiple incoming connections, recall should happen in only one of them at a time. In the NVM, this is implemented via multiplicative gating. One dedicated layer called gts is responsible for this gating mechanism. The gts layer has two gate neurons per connection, one that gates recall and one that gates storage. A gate neuron value of 1 indicates that the operation occurs, and a value of 0 indicates that it does not. Gating patterns are not used as keys and hence are not subject to the orthogonal ±1 restriction. Mathematically, the foregoing equations are revised to incorporate multiplicative gating as follows:


(6) $$\begin{array}{l} {\text{v}}_{\text{r}}^{\left(t+1\right)}=\sigma \left(\underset{\text{C,q}\in C_{\text{r}}}{\sum }u_{\text{C}}^{\left(t\right)}W_{\text{C}}^{\left(t\right)}{\text{v}}_{\text{q}}^{\left(t\right)}\right) \end{array}$$

for gated recall, and for gated storage:


(7) $$\begin{array}{l} W_{\text{C}}^{\left(t+1\right)}=W_{\text{C}}^{\left(t\right)}+\ell_{\text{C}}^{\left(t\right)}\Delta W_{\text{C}}^{\left(t\right)}, \end{array}$$

where $C_{\text{r}}$ is the set of all incoming connections and source layers to destination layer r, and $u_{\text{C}}^{\left(t\right)}$ and $\ell_{\text{C}}^{\left(t\right)}$ are the gate values for associative recall and learning, respectively, in a given connection C. When these gates are zero, there is effectively no recall or storage in the associated connection. The gate values are precisely the corresponding neuron values in gts, i.e.,


(8) $$\begin{array}{l} \left[...,u_{\text{C}}^{\left(t\right)},...,\ell_{\text{C}}^{\left(t\right)},...\right]={\text{v}}_{\text{gts}}^{\left(t\right)}. \end{array}$$

The gate layer determines the currently executing instruction. It has one incoming connection called exe, from a dedicated source layer ipt that represents the current instruction pointer. The instruction pointer is incremented every time-step to advance through a program, using its own self-connection called inc. Each address in program memory is treated as a distinct symbol represented by a distinct activity pattern in ipt, denoted 0_ipt_, 1_ipt_, 2_ipt_, etc. The gate layer evolves according to the same associative recall rule as all other layers, except with a different activation function σ_gts_:


(9) $$\begin{array}{l} {\text{v}}_{\text{gts}}^{\left(t+1\right)}=\sigma_{\text{gts}}\left(W_{\text{exe}}^{\left(t\right)}{\text{v}}_{\text{ipt}}^{\left(t\right)}\right) \end{array}$$

(the recall gate neuron for exe is always 1, and there are no other incoming connections to gts). For σ_gts_ we used an element-wise step function to produce gate values in {0, 1}. In effect, the exe connection remembers which instruction (i.e., gating pattern) is stored at each address in program memory.

A sequence of instructions (i.e., a program) is “compiled” into the NVM by properly initializing $W_{\text{exe}}^{\left(0\right)}$ and $W_{\text{inc}}^{\left(0\right)}$ before program execution begins, so that the proper sequence of instruction pointer addresses and gating operations occurs. Like other connections, these weights are also calculated using the fast store-erase rule, but unlike other connections, they are calculated before (not during) program execution, and their associative learning gates are always 0 during program execution.

Several other dedicated layers and connections are included in the NVM architecture to support sub-routine calls and conditional branching. In this paper we introduce new implementations of these NVM mechanisms that are streamlined from the original versions in Katz et al. (2019). Sections 3.2 and 3.3 describe these new implementations in detail, in the context of robotic block stacking. First, it will be helpful to formalize the version of block stacking we consider in this work.

## 3. Methods

### 3.1. Block Stacking Task

**Figure 3:**
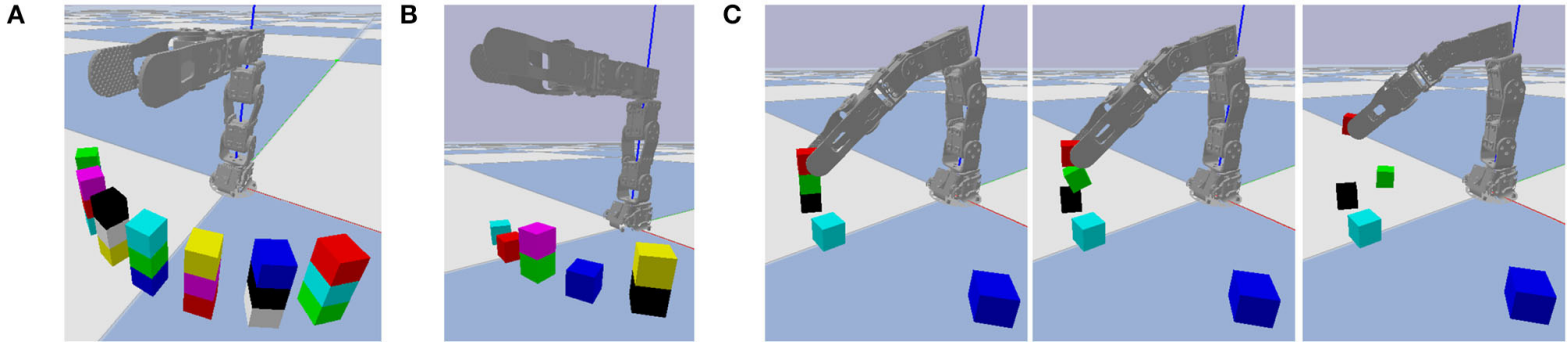
The block stacking environment. **(A)** All possible locations occupied by blocks. **(B)** One state with a typical number of blocks. **(C)** Three video frames of failed grasping, with time increasing from left to right. The robot intends to pick up the red block, but also accidentally lifts the green block off of the black block, causing it to fall on the ground nearby.

**Figure 4:**
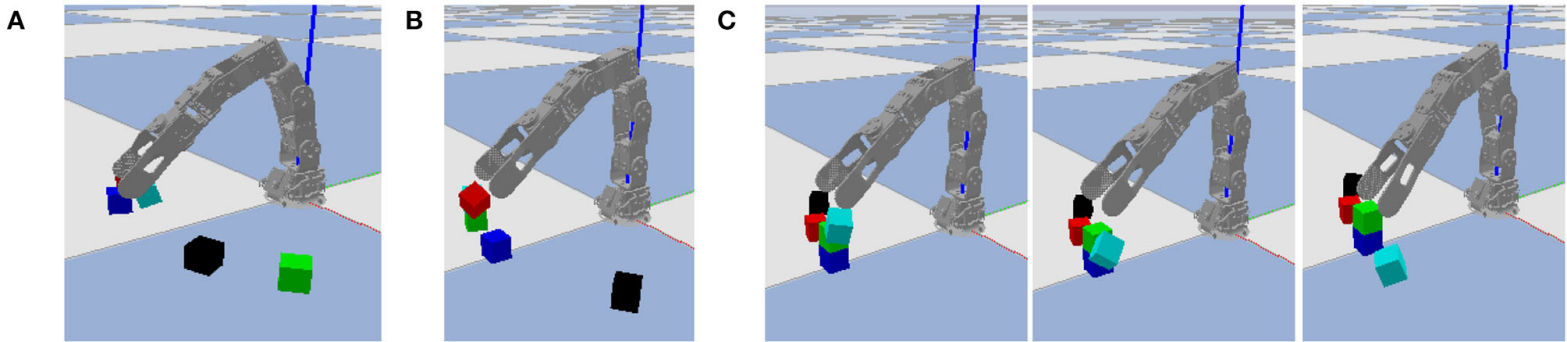
Additional failure cases. **(A)** The cyan block is released in an unstable position and drops due to gravity. **(B)** The gripper moves and knocks the red block from its goal position immediately after releasing it. **(C)** Three video frames where the gripper moves to pick up the cyan block and knocks it in the process.

Similar to the classical “blocks world” domain in automated planning (Nilsson, 1980), we consider a block stacking task in which a set of cubic blocks that are initially stacked in one configuration must be restacked in a new configuration. We predefine seven base locations on which blocks may be stacked into towers, and limit each tower to at most three blocks, as shown in Figure 3A. Whereas, Figure 3A illustrates all possible locations occupied by blocks, our experiments are limited to problem instances with at most seven blocks, stacked in a random configuration, as shown in Figure 3B. This ensures enough base positions on the ground to put every block if needed. One such random configuration is used as initial state, and another as goal state. The robot's objective is to repeatedly pick and place blocks until the actual final state matches the goal state. Even with a perfect high-level plan, low-level motor control errors are possible, as shown in Figure 3C. We use the failure case in Figure 3C as a running example, although other types of failures are also possible as shown in Figure 4.

Each possible block, tower base, and location has its own symbol. The seven block symbols are denoted b_0_ through b_6_, and seven tower base symbols are denoted t_0_ through t_6_. A location symbol of the form (*k*,*h*) denotes the *h*^*th*^ level of the *k*^*th*^ tower, where *k* ∈ {0, …, 6} and *h* ∈ {0, 1, 2} (numbered left-to-right and bottom-to-top). For example, in Figure 3B, the yellow and black blocks are b_1_ and b_6_, and they are stacked on tower base t_6_. The locations of b_1_ and b_6_ are (6,1) and (6,0), respectively. Each block is considered unique; they are not interchangeable in the state or goal descriptions.

States and goals are represented symbolically by a small set of discrete, partial functions: above, right_of, next, loc_of, obj_at, and goal. Their semantics are as follows:

- above[(*k*,*h*)] is the location directly above (*k*,*h*), namely (*k*,*h* + 1).
- right_of[(*k*,*h*)] is the location directly to the right of (*k*,*h*), namely (*k* + 1,*h*).
- next[t_*k*_] is the tower base directly to the right of t_*k*_, namely t_*k*+1_.
- loc_of[b_*n*_] is the location of block b_*n*_, i.e., some location symbol (*k*,*h*) which depends on the current state.
- obj_at[(*k*,*h*)] is the block at location (*k*,*h*), i.e., some block symbol b_*n*_ which depends on the current state.
- goal[b_*n*_] is the block that should be stacked directly on top of b_*n*_ in the goal state, i.e., some other block symbol b_*m*_ (*m* ≠ *n*) that depends on the current goal.

We refer to these partial functions as “mappings.” They have the same semantics as mapping data structures in a high-level programming language, such as HashMaps in Java or dictionaries in Python (hence the square bracket notation). The first three mappings are constant across all states, since the set of possible locations is fixed. The next two are specific to a given state and describe which blocks are at which locations. The last is specific to a given problem instance and describes the goal state. A special symbol nil is returned by these mappings whenever they are evaluated outside their domains (for example, right_of at a right-most location).

As described earlier, a sub-optimal but simple procedure to reach the goal state is to first unstack every tower, so that every block is at a base position, and then restack blocks according to the desired towers in the goal state. To study symbolic and sub-symbolic integration in neurocomputational robotic control, we will program this procedure into the NVM.

### 3.2. Block Stacking With the NVM

**Figure 5:**
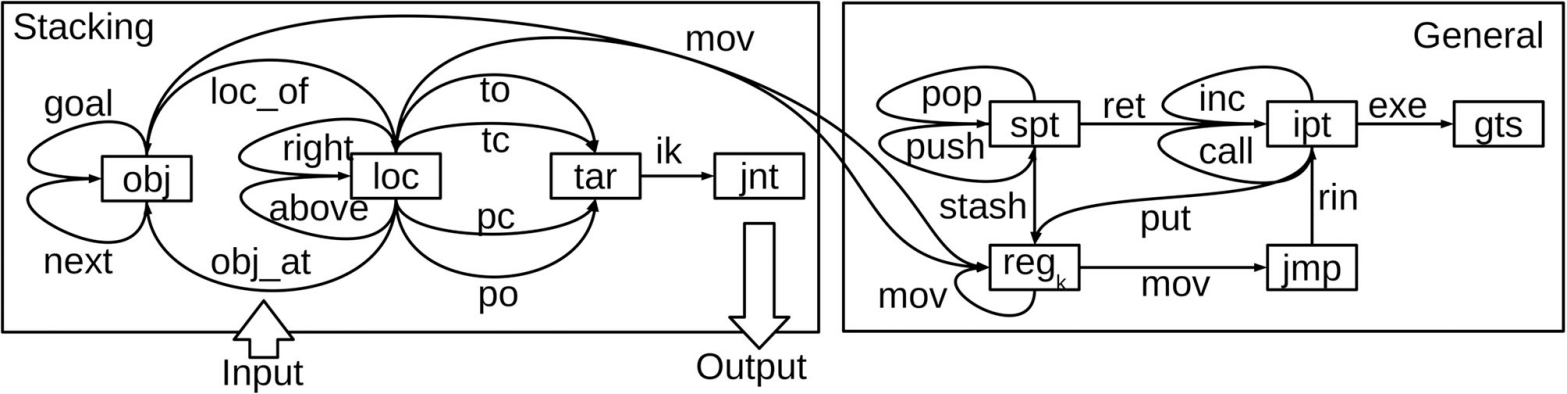
The NVM architecture used for block stacking. Boxes are register layers and line arrows are connection weight matrices, each labeled with their mnemonic. Recall and storage in all connections are gated by gts (gating interactions not shown). Program inputs, consisting of the initial and goal state descriptions, are supplied via initial weights in the obj_at, loc_of, and goal connections. Program outputs, consisting of joint commands, are produced in the jnt layer.

For the block stacking task, we configured the NVM as shown in Figure 5. The boxed registers in the right half of the figure are not specific to block stacking: they are core NVM components needed to execute any program in any application (this is a streamlined version of the NVM with fewer registers than used by Katz et al., 2019). To reduce clutter in the figure, the multiplicative gating effects of gts on all connections in the architecture are not shown. Sub-routine calls/returns and stack increments/decrements, described in more detail below, use the connections call, ret, push, and pop, respectively, in conjunction with a stack pointer register spt. A special flag register called jmp, in conjunction with the rin connection to ipt, supports conditional branching.

The user can configure the NVM with one or more identical general-purpose registers (in this work we used two), denoted collectively by reg_k_ in the figure. The connections labeled mov, between reg_k_ and other layers, are responsible for copying data between registers. The put connection is similar, but used to put literal symbols appearing in the assembly code into a register (hence the connection from the instruction memory pointer ipt). Register data can also be stashed on the stack and restored later, via the stash connection.

The registers in the left half of Figure 5 are specific to block stacking, and communicate with the right half via bidirectional connectivity between obj, loc, and each reg_k_. Object symbols like t_*k*_ and b_*n*_, representing tower bases and blocks, are held in obj (but only one symbol can be held at a time). Likewise, loc holds location symbols. The connections between these layers store the corresponding symbolic mappings from section 3.1 (loc_of, obj_at, etc.), as labeled.

In principle, one could avoid these additional problem-specific connections, by using generic heap memory pointers and registers like those in the original NVM (Katz et al., 2019). The trade-off is that one would need to implement the key-value lookup tables for loc_of, obj_at, etc. in heap memory using NVM assembly code, leading to larger layer sizes and more time-steps of emulation.

The input to the stacking procedure is the state- and goal-specific symbolic information, i.e., the key-value pairs in the loc_of, obj_at, and goal connections. Different block stacking problem instances will involve different key-value pairs in these connections. This symbolic information is converted to synaptic weight values in the corresponding NVM connections, by applying one step of fast store-erase learning to each key-value pair in the mapping at the start of the episode, before running the NVM. Therefore, the sub-symbolic inputs to the NVM are not vectors of initial neural activity, but in fact matrices of initial synaptic weights in these three connections.

The output layer of the NVM is the jnt register layer, which has one real-valued neuron per robotic joint. The vector in this layer changes over time as the NVM emulates the stacking procedure. At any given time-step, it is used as vector of target joint angles for position control. Since the contents of jnt are never used as keys, they are not subject to the orthogonal ±1 restriction, and the activation function for jnt is the identity function, which facilitates real-valued joint angle output.

Individual pick-and-place actions are based on a pre-defined set of end-effector target poses; four per location. Each target pose has a dedicated symbol that can be recalled in the tar register. These poses serve as waypoints during grasp and release motions: **p**erched above the location with gripper **o**pen (po), at the **t**arget location with gripper **o**pen (to), at the **t**arget with gripper **c**losed (tc), or **p**erched above with gripper **c**losed (pc). The corresponding connections are used to select one of these four poses at the current location in loc. For each pose at each location, PyBullet's built-in inverse kinematics routine was used to pre-compute target joint angles that reach the pose. The result is a key-value mapping in which keys represent end-effector poses, and values are corresponding joint angle vectors. This key-value mapping is stored in the NVM ik connection from the tar register to jnt.

**Figure 6:**
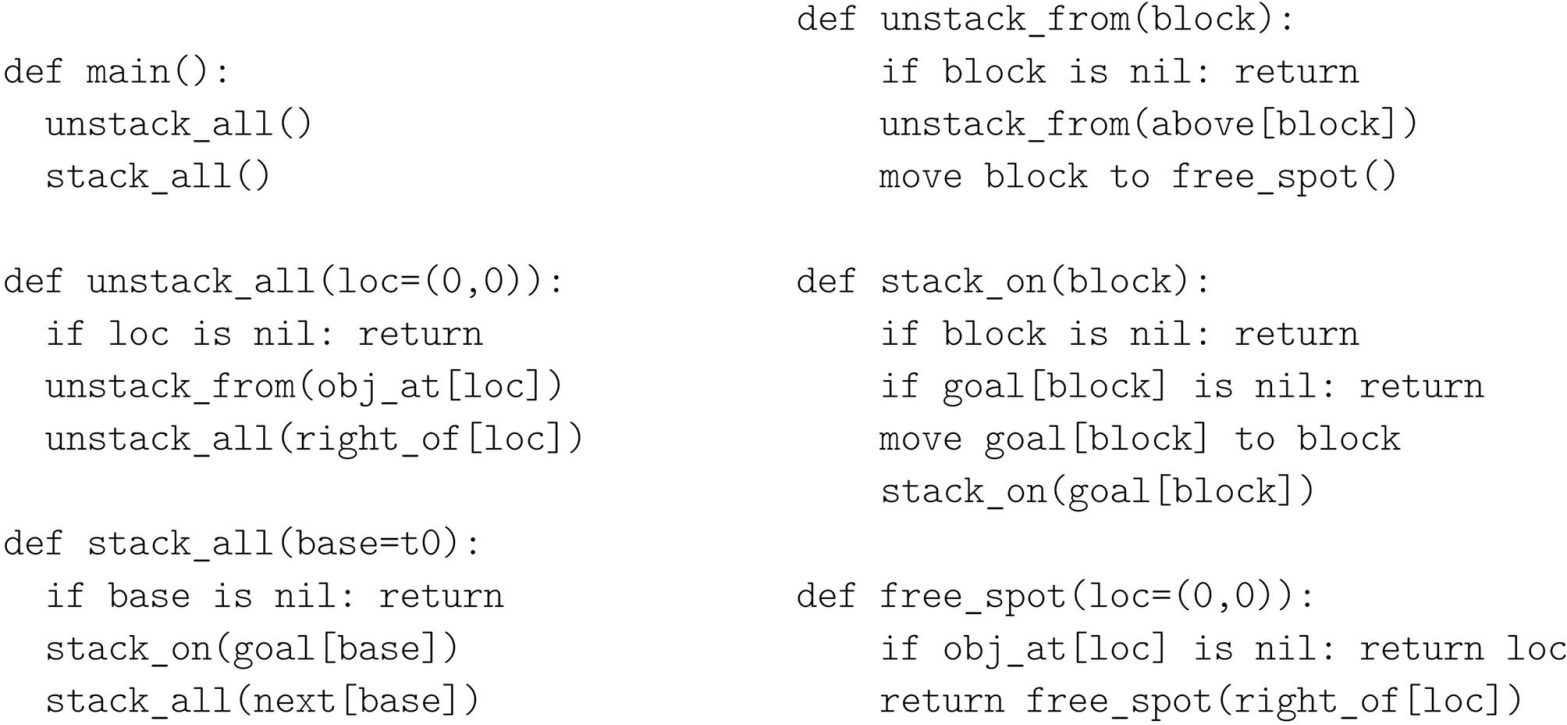
The restacking procedure pseudocode in Python-like syntax. loc=(0,0) and base=t0 are default input values in an initial recursive call. These are implemented in assembly by moving the symbol into a designated register before calling the routine. The “move … to …” commands were implemented by moving a sequence of end-effector poses into the tar register and recalling the corresponding joint angles via the ik connection.

As described earlier, we use a simple two-phased procedure to restack blocks into their goal configuration. First all towers are unstacked in top-down order, moving blocks to free unoccupied tower base positions. Second, all blocks are stacked into their goal positions, one tower at a time, in bottom-up order. This procedure is summarized in high-level pseudocode in Figure 6. All loops are implemented recursively by processing the current location or block, and then invoking a recursive call on the next location or block as appropriate. Two outer loops (unstack_all and stack_all) iterate over towers, and two inner loops (unstack_from and stack_on) iterate over blocks within those towers. One more loop (free_spot) is used to find the next available free spot on the ground, searching for unoccupied tower bases from left to right. NVM assembly does not support input or output variables in sub-routine calls, so we reimplemented this pseudocode in logically equivalent but more verbose assembly code that used registers instead of input/output variables, and one sub-routine for each method defined in the pseudocode.

### 3.3. Block Stacking Execution Trace

This section provides more details on the neurocomputational NVM implementation using an example “execution trace,” i.e., the NVM state at each time-step as it emulates the assembly code for the stacking algorithm. We focus on the free_spot method of the pseudocode in Figure 6, which includes a sub-routine call and conditional branching, to illustrate how these core functionalities are supported by the NVM in the context of block stacking. In this work, we implement conditional branching at the assembly code level, as explained below. This avoids the specialized connectivity and learning rules used for conditional branching in the original NVM (Katz et al., 2019).

**Figure 7:**
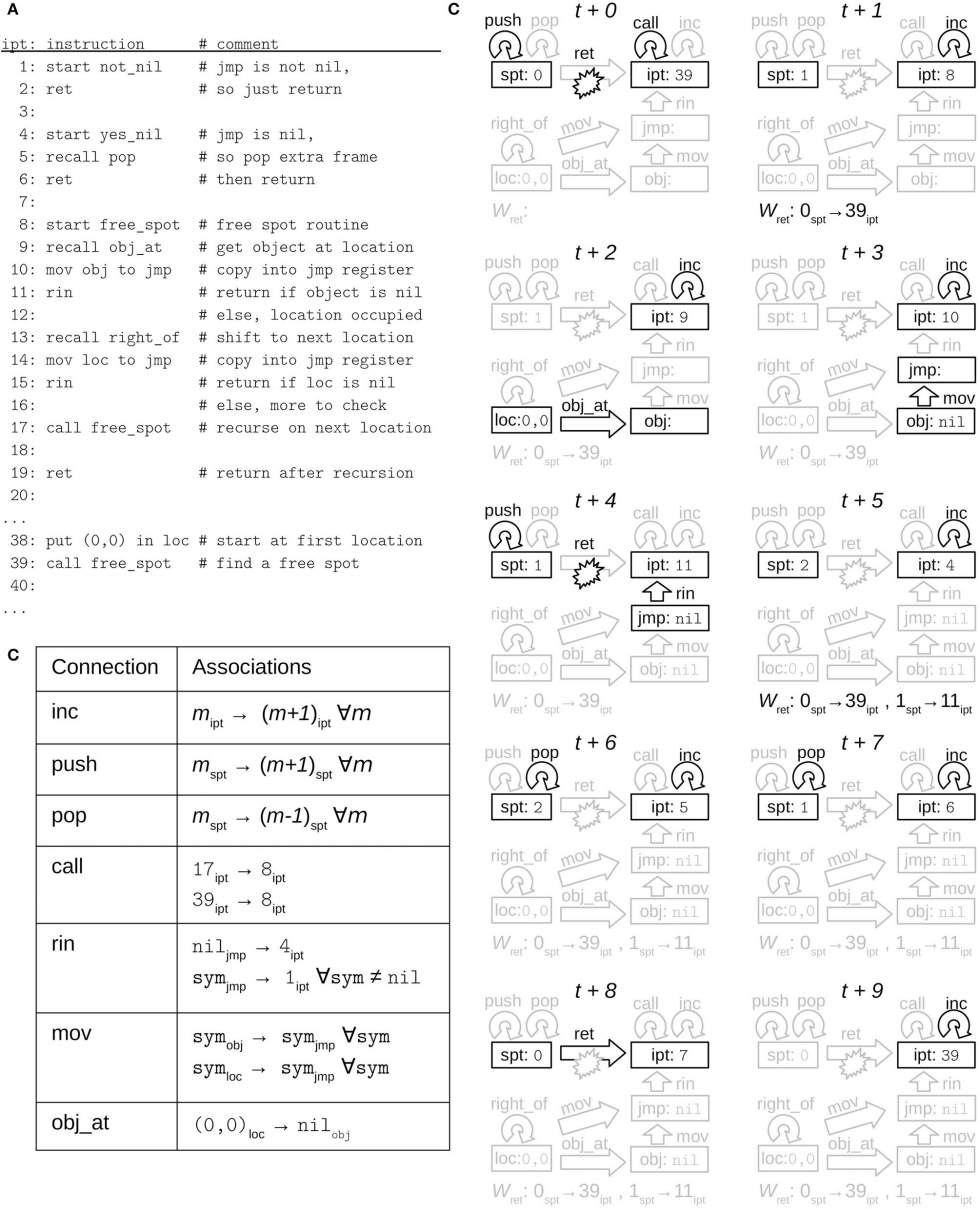
Example NVM execution trace. **(A)** Assembly source code being emulated. **(B)** Initial associations stored when execution begins. **(C)** Gating patterns on the relevant layers and connections (same format as Figure 2) for 10 consecutive time-steps. The associations currently stored in *W*_ret_ are shown below each time-step.

The block stacking assembly program is shown (in part) in Figure 7A. Lines 38–39 put the left-most location in loc and then initiate the recursive free_spot sub-routine, defined on lines 8–20. Lines 9–12 return immediately if the current location is free, and lines 13–16 return immediately if the right-most location has been reached. Otherwise, lines 17–20 recursively process the remaining locations before returning. Here, conditional branching uses a “return if nil” instruction rin, which immediately returns from the sub-routine where it is executed, but only when the nil symbol is present in the flag register jmp.

The rin instruction is implemented by lines 1–7. This code is non-user-facing “firmware” that is compiled when the NVM instance is first constructed, and exposed to the user only via the rin instruction. When nil is present in jmp, the rin instruction calls the sub-routine on lines 4–7; otherwise it calls lines 1–3. The latter is a no-op which simply returns (ret on line 2), but the former pops an additional stack frame, so that it returns to the line where rin's *caller* was called (e.g., 39), not where rin was called (e.g., 11).

Proper emulation requires several associations stored in various connections when execution begins, including but not limited to those in Figure 7B. The inc self-connection on instruction pointer register ipt associates each address in program memory with its successor, to support “incrementing” the instruction pointer during sequential code execution. Similarly, push and pop associate each address in stack memory with its successor and predecessor to support incrementing and decrementing the stack pointer. The call connection associates each line that calls a sub-routine with the starting address of the sub-routine, to support *non*-sequential execution of sub-routine calls. The rin connection from the flag register jmp to ipt supports *conditional* non-sequential execution, depending on the content of jmp, by associating nil with the yes_nil sub-routine, and all other symbols with the not_nil sub-routine. Every pair of registers has a mov connection to support copying data between registers, which associates each symbol with itself (the same symbol can be represented by different activity patterns in different layers). Lastly, obj_at associates each location with the object stored there. In this example, we assume that location (0,0) is currently unoccupied, and therefore associated with nil.

Figure 7C shows the register contents and gating patterns at 10 consecutive time-steps, starting from some time *t* when line 39 is executed. In this example, since the left-most location is unoccupied, there is no recursion after the first call to free_spot, which immediately returns after the rin instruction on line 11 invokes the yes_nil branch on lines 4–7. In more detail, the time-steps proceed as follows:

- *t* + 0: Associative learning in the ret connection saves the current instruction pointer, 39_ipt_, at stack memory address 0_spt_, and associative recall in push increments the stack pointer. Recall in call updates the instruction pointer non-sequentially to line 8_ipt_, where the free_spot sub-routine begins, due to the previously stored call associations in Figure 7B.
- *t* + 1: Recall in inc advances the instruction pointer to the first instruction of free_spot.
- *t* + 2: Recall in obj_at retrieves nil from location (0,0), indicating a free spot.
- *t* + 3: Recall in mov copies nil from obj into the flag register in preparation for conditional branching.
- *t* + 4: Similar to sub-routine calls, the rin instruction saves the current instruction pointer on the stack (via storage in the ret connection) and increments the stack pointer (via push). Associative recall in the rin connection non-sequentially updates ipt to line 4_ipt_, due to nil being present in jmp and the previously stored rin association in Figure 7B.
- *t* + 5: inc advances ipt to the first instruction of the yes_nil firmware routine.
- *t* + 6, 7: Two steps of recall in pop decrement the stack pointer, discarding two stack frames.
- *t* + 8: There is no instruction on line 7, but this line's program memory address in ipt is still used as a key in the exe connection to the gate layer gts. With the stack pointer updated from the previous time-step, associative recall in the ret connection can now be ungated to retrieve 39_ipt_, the program address where free_spot was originally called.
- *t* + 9: inc advances ipt past line 39 now that the free_spot sub-routine call is complete.

If (0,0) were not free, a non-nil block symbol would have moved into jmp, and lines 1–3 would have executed with only one stack pop instead of two. As a result, the ret instruction on line 2 would have returned to line 11 in free_spot rather than line 39, and the search for a free spot would have continued.

We also note that some assembly-level instructions require two or more gating patterns at the neuro-computational level, such as the ret on line 6 that also needed the blank line 7 in time-step *t* + 8. These additional program addresses are inserted automatically by the NVM assembler so that the user does not need to explicitly add them or reason about them.

### 3.4. Validation Metrics

After programming the block-stacking NVM as described above, we empirically validated the effectiveness of the full neurorobotic system using the PyBullet simulation environment. All experiments were performed on an 8-core Intel i7 CPU with 32GB of RAM.

To validate the NVM, we compared it against the non-neural, purely symbolic reference implementation of the VM (RVM), whose execution traces serve as a “gold standard.” We used a large set of problem instances, with total number of blocks ranging from three to seven. For each total number of blocks, 500 independent trials were conducted. In each trial, a random problem instance was generated, with distinct start and goal states. Then the NVM and RVM were each invoked to perform the restacking procedure on the problem instance.

Each initial state and goal in each trial were randomly generated as follows. First, seven empty towers were initialized, one for each base position. Then, blocks were placed on towers one at a time. For each block, its destination tower was chosen uniformly at random from those that did not already contain three blocks, to limit the maximum tower height to three. We chose this method for simplicity, although the resulting distribution of random states is not necessarily uniform. In future work it may be possible to adapt more sophisticated blocks-world sampling methods to our version of block stacking, e.g., Slaney and Thiébaux (2001), to guarantee a truly uniform distribution of states.

The NVM and RVM were both executed on each problem instance, and their performance was compared using three metrics. The first metric is the total number of “ticks,” i.e., the number of machine cycles in the RVM and the number of time-steps in the NVM's recurrent dynamics before completion of the procedure. Since the NVM is intended to perfectly emulate the RVM, the tick counts should be identical for both.

The second metric is total time elapsed during execution, measured in seconds. This metric is loosely correlated with tick counts, but is not exactly the same, since some ticks ungate more connections than others and require more matrix-vector multiplications.

The third metric measures how far the actual final state is from the goal state, at the symbolic level. We refer to this metric as “symbolic distance.” Specifically, we count the number of block pairs (b, b′) where b′ is *supposed* to be on top of b in the goal state, but is *not* on top of b in the actual final state. Small noise in the spatial block positions is ignored, as long as the on-top relation is respected. Letting (*x*_b_, *y*_b_, *z*_b_) denote the spatial coordinates of a block b, as reported by PyBullet at the end of the simulation, we compute the on-top relation as follows:


(10) $$\begin{array}{l} \text{on-top}\left(\text{b}\right)=\underset{{\text{b}}^{\prime }}{\text{argmin }}{\;z}_{{\text{b}}^{\prime }}\\ \text{subject to }z_{\text{b}}<z_{{\text{b}}^{\prime }}\\ \text{ max}\left(\left|x_{\text{b}}-x_{{\text{b}}^{\prime }}|,|y_{\text{b}}-y_{{\text{b}}^{\prime }}\right|\right)<s/2 \end{array}$$

where *s* is the side-length of a block. If there is no b′ satisfying these constraints, we conclude that nothing is on top of b. In other words, we list all blocks (if any) whose (*x, y*) coordinates are sufficiently close to that of b, and select the one whose vertical *z*-position is closest to (but not below) that of b.

We also quantified low-level motor control mistakes mid-way through an episode by defining the following “movement penalty” ρ^(τ)^ at step τ of the PyBullet physics simulation:


(11) $$\begin{array}{l} \rho^{\left(\tau \right)}=\underset{n}{\sum }\|{\dot{\text{p}}}_{{\text{b}}_{n}}^{\left(\tau \right)}\|\cdot \|{\text{p}}_{{\text{b}}_{n}}^{\left(\tau \right)}-{\text{p}}_{\text{gripper}}^{\left(\tau \right)}\|, \end{array}$$

where ${\text{p}}_{{\text{b}}_{n}}^{\left(\tau \right)}=\left(x_{{\text{b}}_{n}}^{\left(\tau \right)},y_{{\text{b}}_{n}}^{\left(\tau \right)},z_{{\text{b}}_{n}}^{\left(\tau \right)}\right)$ is the spatial position of block b_*n*_ at step τ, ${\dot{\text{p}}}_{{\text{b}}_{n}}^{\left(\tau \right)}$ is its velocity, and ${\text{p}}_{\text{gripper}}^{\left(\tau \right)}$ is the position of the gripper (i.e., the point directly in between the two finger-tips where a grasped block should be centered). This formula only penalizes blocks that are moving and not gripped, since $\|{\dot{\text{p}}}_{{\text{b}}_{n}}^{\left(\tau \right)}\|=0$ for stationary blocks, and $\|{\text{p}}_{{\text{b}}_{n}}^{\left(\tau \right)}-{\text{p}}_{\text{gripper}}^{\left(\tau \right)}\|\approx 0$ for a block that is properly gripped, even if it is moving along with the gripper.

Simulation steps are distinct from NVM/RVM tick counts, because multiple steps of physics simulation are performed after sending each joint command. Letting τ_*t*_ denote the physics simulation step at tick *t* of the NVM, we computed a total movement penalty ${\hat{\rho }}^{\left(t\right)}$ associated with the *t*^*th*^ joint command by aggregating the sub-sequence of movement penalties accrued while the joint command is simulated:


(12) $$\begin{array}{l} {\hat{\rho }}^{\left(t\right)}=\overset{\tau_{t+1}}{\underset{\tau =\tau_{t}}{\sum }}\rho^{\left(\tau \right)}. \end{array}$$

### 3.5. Training Process

We conducted the foregoing empirical validation to confirm whether the NVM can properly encode the symbolic information in the RVM, and duplicate its performance. However, there is little reason to simply duplicate what is already possible symbolically, especially given the computational overhead (matrix-vector multiplication, etc.) in the NVM. The main benefit of the NVM is that its neural implementation is amenable to sub-symbolic learning techniques. This motivated us to test whether reinforcement learning (RL) during an additional “practice” phase could boost the NVM's performance, using the connection matrices “compiled” from the RVM as a sophisticated form of weight initialization. We used vanilla policy gradient (VPG) optimization (Williams, 1992; Sutton and Barto, 2018), a classic RL technique, to fine-tune the “compiled” NVM weights. The objective was to minimize the expected symbolic distance at the end of a trial. The expectation was taken with respect to the same random distribution of problem instances used to generate data in section 3.4, except that the number of blocks and bases was fixed at five for computational expediency.

To train the NVM with VPG, we must formalize it as a stochastic policy. The NVM can be viewed as a function


$$\begin{array}{l} \left\{W_{\text{C}}^{\left(T\right)}\right\},\left\{{\text{v}}_{\text{r}}^{\left(T\right)}\right\},...,\left\{W_{\text{C}}^{\left(t\right)}\right\},\left\{{\text{v}}_{\text{r}}^{\left(t\right)}\right\},...,\left\{W_{\text{C}}^{\left(1\right)}\right\},\left\{{\text{v}}_{\text{r}}^{\left(1\right)}\right\}=\\ \text{ NVM}\left(\left\{W_{\text{C}}^{\left(0\right)}\right\},\left\{{\text{v}}_{\text{r}}^{\left(0\right)}\right\}\right) \end{array}$$

where the sets $\left\{W_{\text{C}}^{\left(t\right)}\right\}$ and $\left\{{\text{v}}_{\text{r}}^{\left(t\right)}\right\}$ range over all connections and registers, respectively. The inputs to this function are the initial weights and activities at *t* = 0, and the outputs are all subsequent weights and activities computed by the NVM recurrent dynamics (Equations 6 and 7), until a time *T* when the episode terminates. In particular, some of this function's outputs are the joint angle targets ${\text{v}}_{\text{jnt}}^{\left(t\right)}$ at each time-step which are used to direct the robot. To make the policy stochastic, we use ${\text{v}}_{\text{jnt}}^{\left(t\right)}$ as the mean of a multivariate Normal distribution, and sample actual joint commands θ^(*t*)^ from that distribution:


(13) $$\begin{array}{l} \theta^{\left(t\right)}\sim N\left({\text{v}}_{\text{jnt}}^{\left(t\right)},\epsilon^{2}I\right), \end{array}$$

where *I* is the identity matrix and ϵ is a small scalar (for simplicity, each joint angle is sampled independently with small variance). We fixed ϵ at 0.0174 rad ≈ 1°, which was found to yield a reasonable balance of exploration and exploitation.

For VPG we also require a reward function. One natural candidate is to use symbolic distance at the end of an episode, multiplied by −1 so that smaller distances translate to higher rewards. However, we found that a single reward signal at the end of the episode is too sparse for effective learning. Intermediate rewards throughout the episode were critical for VPG to properly assign credit to the specific joint motions responsible for failure, such as that shown in Figure 3C. For this purpose we also incorporated the per-tick movement penalties (Equation 12). Letting *d*_sym_ denote the symbolic distance at the end of an episode, the complete reward function is:


(14) $$\begin{array}{l} r^{\left(t\right)}=\{\begin{array}{cc} -{\hat{\rho }}^{\left(t\right)} & \text{for }t<T\\ -{\hat{\rho }}^{\left(t\right)}-d_{\text{sym}} & \text{for }t=T \end{array}. \end{array}$$

As explained in section 3.2, $W_{\text{loc\_of}}^{\left(0\right)}$, $W_{\text{obj\_at}}^{\left(0\right)}$, and $W_{\text{goal}}^{\left(0\right)}$ encode the initial and goal states specific to a given problem instance. We view these three matrices collectively as the “state observation” provided to the NVM policy. All other initial weights at *t* = 0 could be used as trainable parameters of the policy. However, we found that NVM program execution is highly sensitive to connection weights used for program instruction memory (right half of Figure 5). Perturbations to these connection weights resulted in episodes where program execution devolved completely, so that no joint commands (and hence reward signals) were generated. Therefore, we limited trainable parameters Ω to all other stacking-specific connections in the left half of Figure 5, namely:


(15) $$\begin{array}{l} \Omega =\left\{W_{\text{next}}^{\left(0\right)},W_{\text{right}}^{\left(0\right)},W_{\text{above}}^{\left(0\right)},W_{\text{to}}^{\left(0\right)},W_{\text{tc}}^{\left(0\right)},W_{\text{po}}^{\left(0\right)},W_{\text{pc}}^{\left(0\right)},W_{\text{ik}}^{\left(0\right)}\right\} \end{array}$$

Since $W_{\text{next}}^{\left(0\right)}$, $W_{\text{right}}^{\left(0\right)}$, and $W_{\text{above}}^{\left(0\right)}$ all encode symbolic information describing the block-stacking environment, this still allowed the NVM to refine its encoding of some symbolic block-stacking knowledge.

Using the foregoing setup, we trained the NVM with VPG for 64 training iterations. In each iteration, we sampled *P* = 16 random problem instances. For each problem instance, we encoded the initial state and goal in $W_{\text{loc\_of}}^{\left(p,0\right)}$, $W_{\text{obj\_at}}^{\left(p,0\right)}$, and $W_{\text{goal}}^{\left(p,0\right)}$, where *p* ∈ {1, …, *P*} indexes the problem instance. We then ran the NVM dynamics to generate a sequence of *T*_*p*_ joint vectors, ${\text{v}}_{\text{jnt}}^{\left(p,t\right)}$, where *t* ∈ {1, …, *T*_*p*_} indexes the NVM ticks. Next, we ran *N* = 16 independent episodes for each problem instance. Each episode used its own independent randomly sampled trajectory of joint commands, $\theta^{\left(p,t,n\right)}\sim N\left({\text{v}}_{\text{jnt}}^{\left(p,t\right)},\epsilon^{2}I\right)$, where *n* ∈ {1, …, *N*} indexes the episode. Each episode was simulated in PyBullet to generate a corresponding sequence of rewards *r*^(*p, t, n*)^. To reduce variance of the policy gradient estimate, we used rewards-to-go *R*^(*p, t, n*)^, and averaged the per-tick rewards-to-go as a baseline *b*^(*p, t, n*)^ (Sutton and Barto, 2018):


(16) $$\begin{array}{l} R^{\left(p,t,n\right)}=\overset{T_{p}}{\underset{k=t}{\sum }}r^{\left(p,k,n\right)} \end{array}$$

(17) $$\begin{array}{l} b^{\left(p,t\right)}=\frac{1}{N}\overset{N}{\underset{n=1}{\sum }}R^{\left(p,t,n\right)} \end{array}$$

These quantities were used to estimate the gradient of expected reward with respect to all trainable parameters Ω, according to the policy gradient theorem (Williams, 1992):


(18) $$\begin{array}{l} \nabla_{\Omega }𝔼\left[\underset{t}{\sum }r^{\left(t\right)}\right]\approx \\ \frac{1}{P\cdot N}\overset{P}{\underset{p=1}{\sum }}\overset{N}{\underset{n=1}{\sum }}\overset{T_{p}}{\underset{t=1}{\sum }}\left(R^{\left(p,t,n\right)}-b^{\left(p,t\right)}\right)\nabla_{\Omega }\log\varphi \left(\theta^{\left(p,t,n\right)}|{\text{v}}_{\text{jnt}}^{\left(p,t\right)},\epsilon^{2}I\right), \end{array}$$

where φ(·|μ, Σ) is the probability density function of N(μ,Σ). We used PyTorch (Paszke et al., 2019) to evaluate ∇_Ω_logφ by backpropagating through all time-steps and layers, except the gate layer, since σ_gts_ used a non-differentiable step function. Ω was updated using the resulting gradient value in conjunction with the Adam optimizer (Kingma and Ba, 2015), with a learning rate of 0.0005 and default values for all other hyper-parameters.

## 4. Results

### 4.1. Empirical Validation

**Figure 8:**
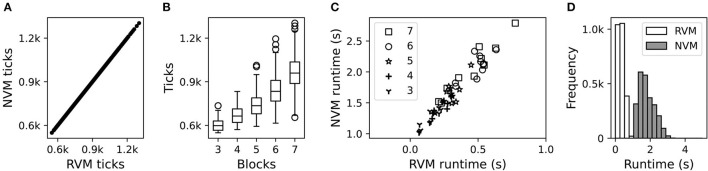
Computational performance of the NVM and RVM. **(A)** Scatter plot of ticks used by the RVM vs. NVM, across all block counts. Each data-point is an independent trial. **(B)** Box plot of the tick distributions (identical for NVM and RVM) for different numbers of blocks. **(C)** Scatter plot of runtime in seconds (s) for the RVM vs. NVM, showing different block counts with different markers. Each data-point is an independent trial. To de-clutter the plot, we only show 10 out of 500 independent trials for each block-count, sub-sampled uniformly at random. **(D)** Histogram of marginal runtime distributions for the RVM (white bars) vs. NVM (gray bars), irrespective of block count.

Our results confirmed that NVM and RVM tick counts are identical (Figure 8A), serving as a sanity check that the NVM was operating correctly. As expected, problem instances with more blocks require more ticks to execute the complete restacking procedure (Figure 8B).

The NVM and RVM were also highly correlated on execution time (Figure 8C), although the absolute runtime in seconds was substantially higher for the NVM (Figure 8D). Unlike its non-neural counterpart, each tick of the NVM involves a large number of matrix operations, so higher runtimes are to be expected. The average RVM clock rate (i.e., ticks per second) was roughly 2,143 Hz. The average NVM clock rate, roughly 465 Hz, was much lower, but still reasonable for near-real-time control of a robotic system.

**Figure 9:**
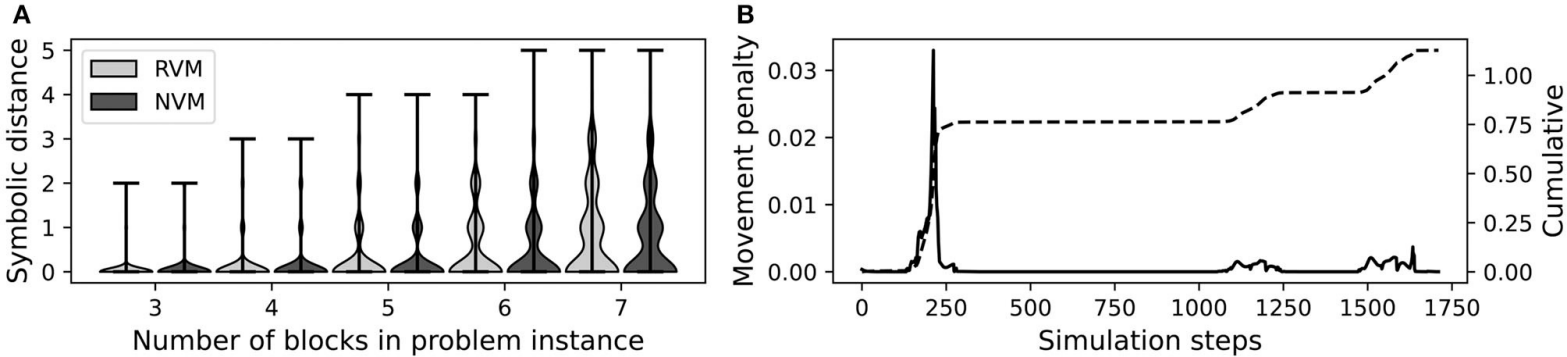
Block stacking performance of the RVM and NVM. **(A)** Violin plots of symbolic distance between actual final state and goal. At each block count (x-axis), we show one violin for the RVM and another for the NVM, as indicated in the legend. The violin plot shows the distribution of symbolic distances over all 500 trials for a given number of blocks. **(B)** Individual (left vertical axis, solid black line) and cumulative (right vertical axis, dashed black line) movement penalties during a failed episode. The spike around simulation step 250 corresponds to the problematic grasp in Figure 3C.

Figure 9A confirms that the NVM performed comparably to the RVM, as measured by symbolic distance. Both versions encountered difficulty as the number of blocks increased. This is expected, as more blocks tend to introduce more chances for low-level motor control mistakes.

Figure 9B plots the individual and cumulative movement penalties during an entire representative episode. The large spike around simulation step 250 corresponds to the problematic grasp shown in Figure 3C. In that example, the green block is supposed to remain stationary while the red block is lifted, but it is moving while not gripped, leading to a large movement penalty.

### 4.2. Improving NVM Performance With Sub-symbolic Fine-Tuning

**Figure 10:**
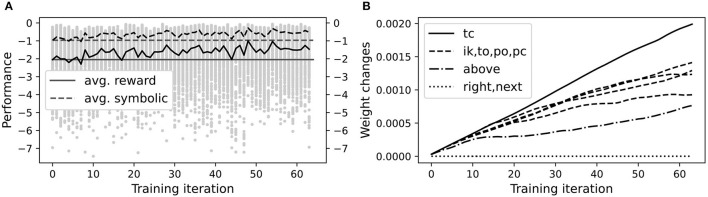
A representative VPG training run. **(A)** Each gray dot shows total reward in one episode of one training iteration. The solid black curve is average total reward over all episodes in a given iteration. The dashed black curve shows average final symbolic distance (multiplied by −1), with movement penalties excluded. Horizontal solid and dashed lines show respective values in the first iteration as a reference point. **(B)** Changes in each trainable weight matrix during training, relative to initial weights, as measured by max norm. In the legend, the labels “tc,” “ik,” etc. are the names of each trainable weight matrix as described in the text. Changes to the right and next weight matrices were non-zero but on the order of 10^−7^.

After validation, we used the reinforcement learning procedure in section 3.5 to fine-tune the symbolic knowledge encoded in the initial NVM weights and check whether performance improved. Five identical, independent runs of the entire learning process were conducted to gauge reproducibility. A typical run is shown in Figure 10A, demonstrating that the NVM can successfully improve its performance through additional practice and reinforcement. To check whether all trainable connections were learning, including $W_{\text{next}}^{\left(0\right)}$, $W_{\text{right}}^{\left(0\right)}$, and $W_{\text{above}}^{\left(0\right)}$, we measured ${\left\|W_{\text{C}}^{\left(i\right)}-W_{\text{C}}^{\left(0\right)}\right\|}_{\infty }$ for each training iteration *i* and trainable connection C, where the max-norm ‖·‖_∞_ is the maximum absolute value over all matrix entries. As shown in Figure 10B, more learning occurs in connections close to the jnt output layer, but other trainable connections experience some degree of optimization. The largest change was in tc, the connection responsible for **c**losing a gripper at its **t**arget location. This is to be expected since the gripper interacts most with the blocks during the actual grasping motion. More interestingly, the above connection changed almost as much as the other layers, suggesting that the network was refining its encoding of on-top relationships to improve performance.

**Figure 11:**
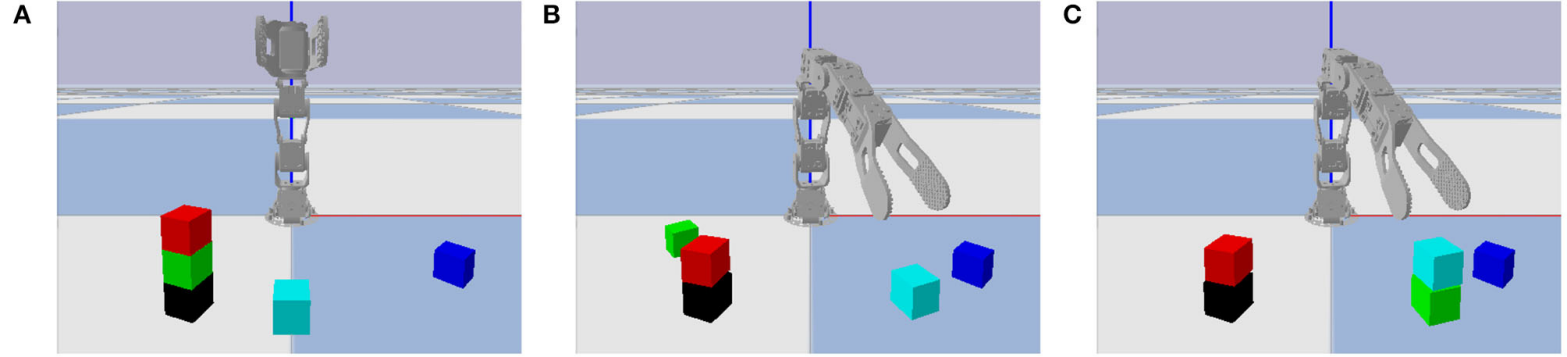
Initial and final states on the failure case in Figure 3C. **(A)** The initial state of the problem instance. **(B)** The final state after RVM execution, with the green block misplaced. **(C)** The final state after NVM execution, using the trained version of the NVM after additional reinforcement learning, with all blocks properly placed.

**Figure 12:**
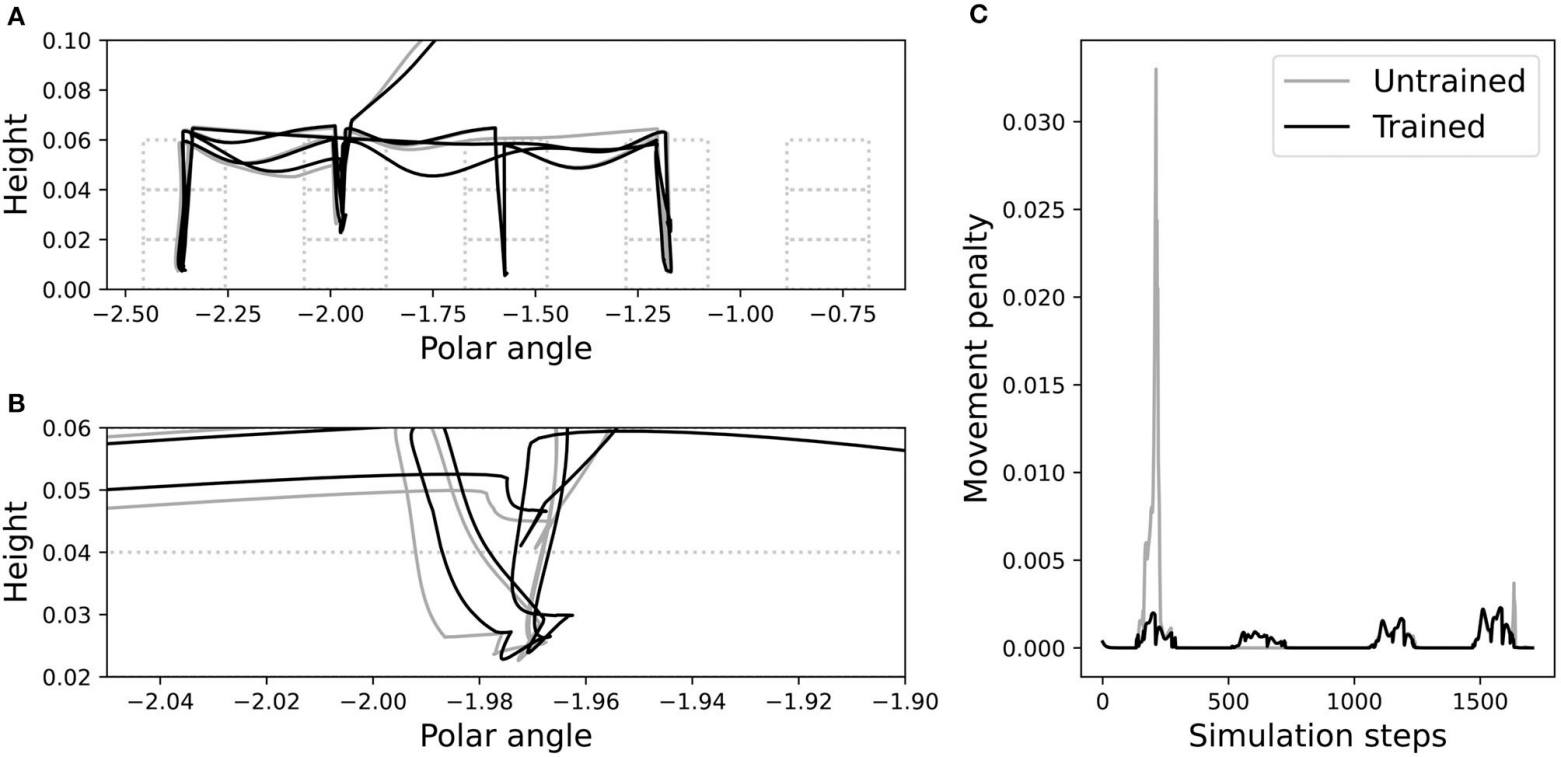
Trajectories before (gray) and after (black) training, in the failure case from Figure 3C. **(A)** The trajectory of the end-effector relative to the blocks, in polar coordinates. Polar angle is the angular distance in radians around the circular arrangement of tower bases. Dashed boxes show the possible block locations. **(B)** A magnified portion of the end-effector trajectory. **(C)** Movement penalties before and after training.

We investigated this further by inspecting performance on the failed problem instance from Figure 3C, before and after training. Figure 11 shows visually that after training, the NVM correctly avoided misplacement of the green block while grasping the red one. The spatial trajectories of the end-effector, when using the RVM vs. the trained NVM, are shown in Figure 12A. It is apparent that the largest change after training is in the vertical direction, corroborating the change observed in above. In particular, magnifying the trajectories around the problematic grasp point (Figure 12B) illustrates that the trained NVM positioned the gripper slightly higher when picking up blocks, which explains how it avoided accidental interaction with the green block. Accordingly, Figure 12C confirms that movement penalties were reduced during the successful execution after training.

**Figure 13:**
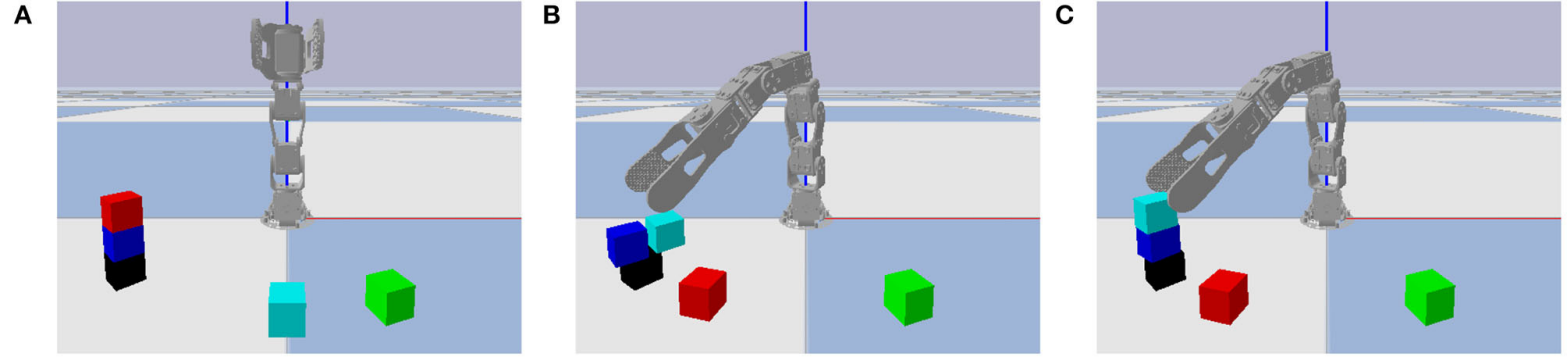
Same format as Figure 11 with a different failure case. After unstacking the red block in the initial state **(A)**, the RVM releases the cyan block in an unstable position and it falls due to gravity **(B)**, but the trained NVM releases it in a stable position **(C)**.

Improved NVM performance was not limited to the one failure case shown in Figure 3C. Manual inspection showed that the trained NVM also improved on other instances with the same type of failure (displacing one block while grasping another), as well as different types of failure (releasing a block in an unstable position), as shown in Figure 13. Some failure types, such as knocking over blocks at the top of the towers while moving, were not fully addressed by the trained NVM. However, as our results show, average performance did improve over the entire problem distribution, which includes all failure cases. Since training does not appear to have converged by iteration 64, it is possible that additional training iterations would further improve performance on all failure cases.

**Figure 14:**
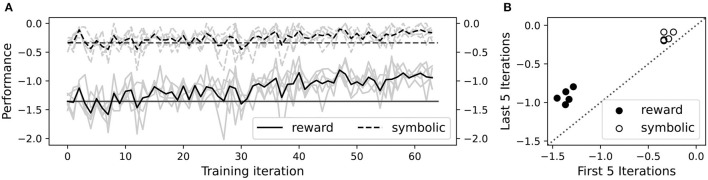
Reproducible performance improvement on five independent training runs. **(A)** Average total rewards including movement penalties (solid curves), and negative symbolic distance excluding movement penalties (dashed curves), during training. Each gray curve plots the per-iteration average over all episodes for one independent training run. Each black curve is the average over all training runs. Horizontal solid and dashed lines show respective values in the first iteration as a reference point. **(B)** Scatter plot of average total reward (black circles) and negative symbolic distance (white circles), in the first five vs. last five iterations of training. There are two circles per independent training run, one black and one white. The dashed line corresponds to no change in performance.

The foregoing performance improvements were reproducible, as shown in Figure 14. All five independent repetitions of the training experiment led to substantial improvement in average reward (including movement penalties), as well as modest improvement in average final symbolic distance (excluding movement penalties). Average symbolic distance did not show dramatic improvement on an absolute scale, but it was already fairly close to zero before training due to the reasonably effective procedural knowledge compiled into the NVM. On a percentage scale, training reduced symbolic distance by over 50% on average. Each independent training run took roughly 2.5 h to complete on our 8-core Intel i7 CPU workstation.

## 5. Discussion

We have shown that a high-level symbolic procedure for blocks world problems can be compiled into a purely neural system, the NVM, to effectively control a simulated robotic manipulator. The NVM precisely emulates the execution traces of a non-neural reference implementation, and achieves a “clock rate” near 465 Hz, which is suitable for robotic control. Moreover, after programming the NVM with symbolic knowledge, its performance can be further improved using reinforcement learning on its sub-symbolic neural substrate. This demonstrates that programmable neural networks supporting symbolic processing, like the NVM, are a viable approach for integrating high- and low-level robotic control.

In future work, this approach should be tested in more natural environments than simulated block stacking. This includes objects with more real-world relevance than blocks, such as tools and household products. It also includes more varied and complex tasks, such as assembly and maintenance problems, or assisted living, disaster recovery, and robotic surgery scenarios (Qi et al., 2021). Furthermore, the validation should include physical robotic hardware in the real world, more sophisticated (e.g., multi-fingered) end-effectors, and other robotic form factors such as humanoids and other mobile manipulators.

Our present system is missing some important elements which would be needed for implementation on a physical robot. In particular, the system should be extended with closed-loop feedback control and a robust sensing sub-system. The sensing sub-system could include visual camera input, haptic feedback, and proprioceptive information such as joint angles, velocities, torques, and temperatures. The sensing sub-system would need to be designed and trained in such a way that its output layer activity is compatible with NVM layer activities. For example, one could train a classifier to identify which object is present in a given region of the visual field, and the classifier's output layer activity could be transformed via a single linear connection into a ±1 pattern used by the NVM obj layer. This kind of mechanism would maintain a purely neural system and transmit location occupancy data from visual input to the NVM. One could also add additional connections directly from the visual system to the NVM jnt layer. This way, cognitive-level joint directives coming from the NVM could be biased by lower-level visual feedback. More work is needed to refine these ideas and engineer an NVM-based controller for a physical robot.

Our present system also lacks several advanced automated planning features such as conditional planning, and planning with sensing, faults, and monitoring. Once a visual system is in place to support sensing and monitoring, several such planning features could be integrated by increasing the sophistication of the procedural knowledge compiled into the NVM. That is, the simplistic block stacking algorithm we programmed into the NVM (Figure 6) could be replaced with a more general and advanced planning algorithm that regularly checks sensory input during execution and replans accordingly when needed.

One more limitation of the present work is the significant computational expense and poor sample efficiency of the VPG-based reinforcement learning. Since our primary goal was to demonstrate that improvement with practice was possible, we limited the training time for computational expediency, but longer training could lead to larger performance boosts. Furthermore, employing modern state-of-the-art robotic RL techniques, such as PPO (Schulman et al., 2017) or SAC (Haarnoja et al., 2018), will likely further improve performance and efficiency, extending the NVM's reach to more complex tasks and robotic platforms.

Lastly, there are several opportunities to leverage the NVM's underlying neural implementation that should be explored further. One possibility is to enhance its biological realism in future design iterations, so that it can both inform, and be informed by, biological neural systems (e.g., humans) performing similar tasks. Given the connection between backpropagation and more biologically plausible, gradient-free contrastive Hebbian learning (Xie and Seung, 2003), gradient-free analogs of policy optimization may be possible in the NVM, especially considering that its fast-weight updates are already Hebbian in nature. Another possibility is to explore the NVM's explainability in more depth. Despite its purely neural substrate, the compatibility with human-readable declarative and procedural knowledge may facilitate more interpretable robotic behavior, potentially even after reinforcement-based fine-tuning.

## Data Availability Statement

The raw data supporting the conclusions of this article will be made available by the authors, without undue reservation.

## Author Contributions

GK was the primary author of the manuscript and code in this work. Akshay assisted with the implementation of the simulation environment and proof-reading the manuscript. GD assisted with the design of the NVM and proof-reading the manuscript. RG and JR assisted with guiding the research direction and proof-reading the manuscript. All authors contributed to the article and approved the submitted version.

## Funding

This work was supported by ONR award N00014-19-1-2044.

## Conflict of Interest

The authors declare that the research was conducted in the absence of any commercial or financial relationships that could be construed as a potential conflict of interest.

## Publisher's Note

All claims expressed in this article are solely those of the authors and do not necessarily represent those of their affiliated organizations, or those of the publisher, the editors and the reviewers. Any product that may be evaluated in this article, or claim that may be made by its manufacturer, is not guaranteed or endorsed by the publisher.

## Footnotes

^1^ https://github.com/garrettkatz/poppy-muffin/tree/master/pybullet/tasks/pick_and_place
